# Modulating cell proliferation by asymmetric division: A conserved pattern in the early embryogenesis of nematode species

**DOI:** 10.17912/micropub.biology.001006

**Published:** 2024-03-04

**Authors:** Guoye Guan, Ce Luo, Lei-Han Tang, Chao Tang

**Affiliations:** 1 Center for Quantitative Biology, Peking University; 2 South Bay Interdisciplinary Science Center, Songshan Lake Materials Laboratory; 3 Department of Physics, Hong Kong Baptist University; 4 Current Address: Department of Systems Biology, Harvard Medical School; 5 Current Address: Department of Data Science, Dana-Farber Cancer Institute; 6 Institute of Computational and Theoretical Studies, Hong Kong Baptist University; 7 State Key Laboratory of Environmental and Biological Analysis, Hong Kong Baptist University; 8 Peking-Tsinghua Center for Life Sciences, Peking University; 9 School of Physics, Peking University

## Abstract

In the early stage of the nematode *Caenorhabditis elegans* embryogenesis, the zygote divides asymmetrically into a symmetric fast lineage and an asymmetric slow lineage, producing 16 and 8 cells respectively almost at the same time, followed by the onset of gastrulation. It was recently reported that this cell division pattern is optimal for rapid cell proliferation. In this work, we compare the cell lineages of 9 nematode species, revealing that this pattern is conserved for >60 million years. It further suggests that such lineage design has an important functional role and it might speed up embryonic development in the nematode kingdom, not limited to *C. elegans* , and independent of the maternal-zygotic transition dynamics.

**Figure 1. Embryonic cell lineage trees of 9 nematode species up to the developmental stage before gastrulation onset:**
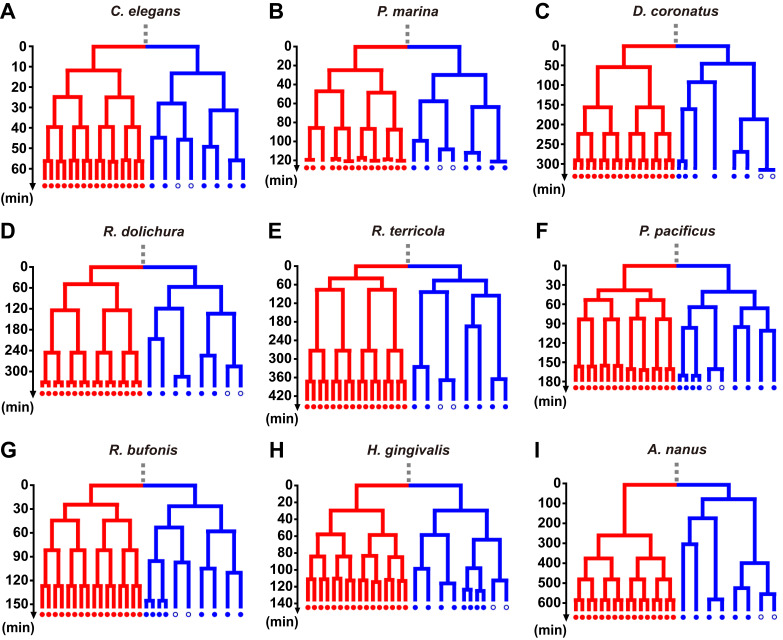
The symmetric and asymmetric lineages are positioned on the left and right in each diagram, and colored in red and blue, respectively; here, the symmetric and asymmetric lineage refers to the differentiation of cell division timing regardless of the differentiation of other cellular properties like cell fate. Among each pair of sister cells within the symmetric or asymmetric lineage, the one with a shorter cell cycle length is placed on the left while the other is on the right; cell cycle lengths are represented by the size of the vertical bars, with an actual timescale located near the lineage of each nematode species. In each nematode species, there are two intestinal precursor cells pre-programmed for gastrulating movement and marked with open circles, except (G) *R. bufonis* , in which at the end of the illustrated lineage the two intestinal precursor cells marked with open circles will undergo one more round of cell divisions then the four newborn progenies will proceed gastrulating movement immediately (see Extended Data) (Spieler and Schierenberg, 1995); all other cells at the end of the illustrated lineage are indicated with dots.

## Description

Cell lineage is the developmental history of a cell, starting from the zygote. This is often supplemented with information about the cell's cycle length, fate, and spatial position (Venuti and Jeffery, 1989; Salvador-Martínez et al., 2019). It can comprehensively characterize the multicellular developmental procedure, spanning from cellular to organismic scales within the system (Sulston, 1976; Sulston and Horvitz, 1977) . So far, many molecular mechanisms responsible for cell lineage programming in different scenarios have been unveiled by genetic approaches (Ho et al., 2015; Wu et al., 2023) . In the last decade, increasing attention has been paid to the developmental fitness of the cell lineage program (Itzkovitz et al., 2012; Fickentscher et al., 2016; Tian et al., 2020; Guan et al., 2021a). A focal point of these studies is the early embryogenesis of nematode *Caenorhabditis elegans* ( Fig. 1A ), which is highly conserved within the *Caenorhabditis* genus for 20 million years (Sulston et al., 1983; Zhao et al., 2008; Memar et al., 2019; Guan et al., 2021b). For example, the cell divisions synchronized in separate groups and with distinct orders could coordinate the cell movements in concert, ensuring the spatial precision and robustness of a *C. elegans* embryo (Fickentscher et al., 2016; Tian et al., 2020) . Remarkably, recent mechanical simulations found that the increase in cell number during *C. elegans* embryonic cell proliferation, which is determined by the cell lineage program, enhances the lateral pressure between neighboring cells and induces mechanical instability in their spatial architecture, consequently triggering and facilitating cell internalization, namely gastrulation (Nance and Priess, 2002; Nance et al., 2005; Miao et al., 2022) ; this pressure increase and cell rearrangement due to spatial confinement match the previous experimental observation that gastrulation is defective when the chitinous eggshell and vitelline membrane are removed (Schierenberg and Junkersdorf, 1992) ; similar mechanical instability that drives cell internalization or tissue invagination was also proposed in other organisms, like *Drosophila* and *Hydra* (Guo et al., 2022; Su et al., 2023) . Furthermore, another recent theoretical study pointed out that the *C. elegans* embryonic cell lineage designated in reality can significantly shorten the time to reach the cell number prior to gastrulation ( *i.e.* , the 24-cell stage); this is achieved through asymmetric volume segregation during the cytokinesis of the zygote, resulting in one fast-dividing and one slowly-dividing blastomere that produces 16 and 8 cells, respectively, in near simultaneity (Nance and Priess, 2002; Nance et al., 2005; Guan et al., 2021a). These findings are in line with the previous discovery that a global transition from synchronous to asynchronous cell divisions is employed to minimize the developmental time of mouse intestinal crypt (Itzkovitz et al., 2012) . The above two *C. elegans* studies raise the possibility that cell lineage programming plays a pivotal role in adjusting and even minimizing the time to reach a specific cell number crucial for certain reasons, exemplified by gastrulation, in theory. However, it remains unknown whether such an optimization strategy based on physical rules ( *i.e.* , separating two sublineages with different cleavage paces to reach the cell number required by mechanical instability or gastrulation quickly) is used to accelerate the embryonic development in other nematode species, considering that their cell lineage patterns and/or regulations could be substantially different from each other (Laugsch and Schierenberg, 2004) .

In this study, a total of 45 nematode species are surveyed in published literature, among which 10 species have complete and clear information recorded, including both the cell number before gastrulation onset and timing of all cell divisions that take place from the 2-cell stage to that moment (see Extended Data). Here we focus on the 9 nematode species ( Fig. 1 ) spreading from Clade 9 to Clade 11 (predicted and defined by phylum-wide analysis of small-subunit ribosomal DNA) considering their evolutionary continuity (Holterman et al., 2006) , *i.e.* , *Caenorhabditis elegans* ( Fig. 1A ) (Guan et al., 2019) , *Pellioditis marina* ( Fig. 1B ) (Houthoofd et al., 2003) , *Diploscapter coronatus* ( Fig. 1C ) (Lahl et al., 2009) , *Rhabditis dolichura* ( Fig. 1D ) (Laugsch and Schierenberg, 2004) , *Rhabditis terricola* ( Fig. 1E ) (Laugsch and Schierenberg, 2004) , *Pristionchus pacificus* ( Fig. 1F ) (Vangestel et al., 2008) , *Rhabdias bufonis* ( Fig. 1G ) (Spieler and Schierenberg, 1995) , *Halicephalobus gingivalis* ( Fig. 1H ) (Houthoofd and Borgonie, 2007) , and *Acrobeloides nanus* ( Fig. 1I ) (Laugsch and Schierenberg, 2004; Schulze and Schierenberg, 2009) ; the excluded one, *Plectus sambesii* , lies in Clade 6 and is far from the others (Schulze and Schierenberg, 2011; Schulze et al., 2012) . Concerning the synchronization and asynchronization of cell division timings, the cell lineage of the nematode species considered always has a half proceeding symmetric cell divisions and another half proceeding asymmetric cell divisions; note that the symmetric and asymmetric cell division here refers to the differentiation of cell division timing regardless of the differentiation of other cellular properties like cell fate. Interestingly, these nematode species exhibit different modes in their cell cycle length patterns before gastrulation onset (see Extended Data). For example, the cell cycle lengths in *C. elegans* keep increasing globally ( Fig. 1A ), while the cell cycle lengths in the symmetric and asymmetric halves of the lineage in *A. nanus* are decreasing and increasing respectively ( Fig. 1I ); moreover, the cell cycle lengths in some species can transit from one mode to another, for instance, the cell cycle lengths in the symmetric half of the cell lineage in *D. coronatus* first increase and then decrease ( Fig. 1C ) (see Extended Data). Previous research suggested that those patterns are governed by a critical process named maternal-zygotic transition, during which the maternal supply and zygotic production on cell-cycle-related factors together mediate the cell cycle lengths (Laugsch and Schierenberg, 2004) . The differential cell cycle lengths are also attributed to another independent factor, the unequal volume segregation ratio during cytokinesis, which unevenly allocates the cell-cycle-related factors as well as other molecular contents and influences the overall cleavage pace of multiple generations in a sublineage (Arata et al., 2015; Fickentscher et al., 2018) .

Intriguingly, although the cell cycle length patterns prior to gastrulation are diverse because of the different underlying regulatory mechanisms, those nematode species still share a common program for cell number control. First, the zygote generates a fast-dividing and a slowly-dividing lineage. Second, these two lineages produce ~16 and ~8 cells almost simultaneously. Third, the gastrulation onset is right after this stage of 24±2 cells (see Extended Data). Note that the separation time between *C. elegans* and *P. pacificus* was at least 60 million years ago according to (Cutter, 2008; Dieterich et al, 2008; Rota-Stabelli et al., 2013; Prabh et al, 2018; Prabh and Rödelsperger, 2022), indicating a highly conserved and stable cell lineage pattern just before gastrulation onset over evolution. The simplest case seems to be the one in *C. elegans* , which has globally decreasing cell cycle lengths as observed *in vivo* and has been demonstrated to be able to shorten the time to reach the 24-cell stage followed by mechanical instability and gastrulation as discovered *in silico* (Guan et al., 2021a; Miao et al., 2022) ; the pattern similarity between *C. elegans* and the other 8 nematode species suggests a strong functional role of modulating cell proliferation by asymmetric division of zygote, which could be to reach quickly the cell number required for gastrulation ( Fig. 1 ). Besides, recent thorough experiments have uncovered the essentiality of the asymmetric division in *C. elegans* zygote for successful embryogenesis, through controlling a series of developmental properties ( *i.e.* , cell cycle length, cell fate, cell position, and cell division orientation) (Jankele et al., 2021) ; we speculate that this essentiality and the relevant mechanisms might also be underlying the other nematode species, in consideration of their conserved pattern ( Fig. 1 ).

In summary, we present cell lineage patterns up to the moment before gastrulation onset quantified from previous literature across 9 nematode species. These patterns exhibit an overall conserved feature as well as diversity in details ( Fig. 1 ). This data can be used in many research topics. In addition to the representative patterns shown in Fig. 1, the cell lineage pattern of some nematode species can be changed to some extent when the creature is developing at different temperatures ( *e.g.* , *R. dolichura* and *R. terricola* ) or in different environments ( *e.g.* , *R. bufonis* ) (see Extended Data), where the division timings of particular cells are changed; in this theme, both experimental observation and theoretical simulation have studied the role of cell division timing program in coordinating cell movement and subsequent cell-cell contact in *C. elegans* (Ho et al., 2015; Wong et al., 2016; Fickentscher et al., 2016; Tian et al., 2020; Eisenmann et al., 2005; Priess et al., 2005) . It would be interesting to investigate how the external conditions mentioned above switch the consequential variable cell division timing programs and whether the cell arrangement and contact-based signaling transduction ( *e.g.* , Notch signaling) are adapted to the varied programs in various nematode species and under various external conditions (Priess, 2005) . Unlike the developmental procedures artificially perturbed by RNAi treatment and laser ablation (Xiao et al., 2022; Kuang et al., 2022) , the ones in nematode species beyond *C. elegans* also end up viable individuals. Thus, the data provided in this study could be useful for building an overall blueprint about how genetic and physical regulations jointly construct an eutelic nematode with cell-level developmental accuracy. It's worth noting that, the developmental procedures depicted in their original literature are measured by different monitoring equipment ( *e.g.* , differential interference contrast microscopy for *P. marina* and confocal microscopy for *C. elegans* ) (Houthoofd et al., 2003; Guan et al., 2019) , in different sample sizes ( *e.g.* , 3 for *R. dolichura* and 222 for *C. elegans* ) (Laugsch and Schierenberg, 2004; Guan et al., 2019) , and at different time scales ( *e.g.* , minute for *H. gingivalis* and hour for *D. coronatus* ) (Houthoofd and Borgonie, 2007; Lahl et al., 2009) , higher accuracy of the collected information might need to be realized by employing the same measurement pipeline with the highest standard as possible, for verifying the data of the cell cycle length patterns and gastrulation onsets summarized in this paper and ensuring the reliable acquisition of the division sequence between particular cells. In the future, more quantitative experimental tools and observations ( *e.g.* , cell position and cell volume) may be needed to achieve a better description of the embryogenesis in different circumstances, and this description is actually a much larger and alternative “parameter space” where a developing nematode embryo can survive.

## Methods

***Quantitative measurement of cell division timing***

The cell division timing was measured by drawing a vertical line from the root of the cell lineage tree plotted in the literature to the end of the branch of a cell, with the aid of the software *ImageJ* (Rasband, 1997-2018; Abramoff et al., 2004; Schneider et al., 2012) . Referring to the time axis plotted in the literature, the length of the line drawn in the software *ImageJ* was transformed into a duration with an actual time unit, minute (see Extended Data).

***Determination of timing prior to gastrulation***

Usually, there are different descriptions of the timing related to nematode gastrulation, including the one prior to gastrulation (Lahl et al., 2009) and the one when gastrulation starts (Vangestel et al., 2008) . For simplicity, we uniformly choose the timing prior to gastrulation as the object of study in this paper (see Extended Data). If only the exact stage of gastrulation onset is given in the literature with a specified cell number, we refer to the cell lineage pattern and search its last stage with a distinct and unambiguous cell number as the timing prior to gastrulation. As exemplified by *C. elegans* , its gastrulation begins at the 26-cell stage so its timing prior to gastrulation is the 24-cell stage when the MS2 cells haven't divided yet (Nance and Priess, 2002; Guan et al., 2019) .

***Information filtering of multiple nematode species***

For each literature scanned, the mentioned nematode species with searchable information on its Clade, Class, Order, Family, and Genus was considered. Among the nematode species, the ones with a clear cell lineage tree plotted in the literature, which starts no later than the 2-cell stage and lasts beyond the moment of gastrulation onset, were picked up. Note that the description of the timing of gastrulation onset is required, as it is a developmental landmark of interest and has been reported to be correlated with the increase of cell number, under the manipulation of multicellular mechanics (Nance and Priess, 2002; Nance et al., 2005; Miao et al., 2022) . Only the nematode species that satisfies all the criteria above is used for plotting a cell lineage tree in Fig. 1 (see Extended Data).

***Temporal normalization of different cell lineage***

Different nematode species have distinct developmental speeds. For example, starting from the 2-cell stage, the time *A. nanus* takes to the 24-cell stage (~583 min) is one magnitude order longer than that *C. elegans* takes (~57 min) (see Extended Data). To compare the 9 nematode species on a consistent time scale, we set the developmental pace of *C. elegans* as a reference for cell lineage tree plotting. For the nematode species with information recorded since the division of the zygote, its duration from the first cleavage to the appearance of the 24-cell stage is linearly scaled to the one in *C. elegans* , along with the subsequent lineage structure. For the nematode species without information recorded until the division of AB cell ( *i.e.* , the founder cell of the fast lineage), its duration from the end of the 2-cell stage to the appearance of the 24-cell stage is linearly scaled to the one in *C. elegans* , along with the subsequent lineage structure; the duration of the 2-cell stage ( *i.e.* , the cell cycle length of AB cell) is determined by setting the duration ratio of the 2-cell stage to 3-cell stage the same as the one in *C. elegans* .

## Extended Data

Description: Trends in cell cycle length, and the available quantitative information on cell number before gastrulation onset as well as number of gastrulating cells in different nematode species.. Resource Type: Dataset. DOI: 10.22002/exj88-a4k98

Description: Cell division timing in different nematode species.. Resource Type: Dataset. DOI: 10.22002/2meeq-a2a35

Description: Matlab code for cell lineage tree plotting.. Resource Type: Dataset. DOI: 10.22002/h3b8s-9xd69
